# Reactivity to low‐flow as a potential determinant for brachial artery flow‐mediated vasodilatation

**DOI:** 10.14814/phy2.12808

**Published:** 2016-06-22

**Authors:** Kunihiko Aizawa, Salim Elyas, Damilola D. Adingupu, Francesco Casanova, Kim M. Gooding, W. David Strain, Angela C. Shore, Phillip E. Gates

**Affiliations:** ^1^ Diabetes and Vascular Medicine Research CentreUniversity of Exeter Medical SchoolExeterUK; ^2^ NIHR Exeter Clinical Research FacilityExeterUK; ^3^ Royal Devon and Exeter NHS Foundation TrustExeterUK

**Keywords:** Brachial artery, cardiovascular disease, endothelial function, shear stress, ultrasound

## Abstract

Previous studies have reported a vasoconstrictor response in the radial artery during a cuff‐induced low‐flow condition, but a similar low‐flow condition in the brachial artery results in nonuniform reactivity. This variable reactivity to low‐flow influences the subsequent flow‐mediated dilatation (FMD) response following cuff‐release. However, it is uncertain whether reactivity to low‐flow is important in data interpretation in clinical populations and older adults. This study aimed to determine the influence of reactivity to low‐flow on the magnitude of brachial artery FMD response in middle‐aged and older individuals with diverse cardiovascular risk profiles. Data were analyzed from 165 individuals, divided into increased cardiovascular risk (CVR:* n* = 115, 85M, 67.0 ± 8.8 years) and healthy control (CTRL:* n* = 50, 30M, 63.2 ± 7.2 years) groups. Brachial artery diameter and blood velocity data obtained from Doppler ultrasound were used to calculate FMD, reactivity to low‐flow and estimated shear rate (SR) using semiautomated edge‐detection software. There was a significant association between reactivity to low‐flow and FMD in overall (*r *=* *0.261), CTRL (*r *=* *0.410) and CVR (*r *=* *0.189, all *P *<* *0.05) groups. Multivariate regression analysis found that reactivity to low‐flow, peak SR, and baseline diameter independently contributed to FMD along with sex, the presence of diabetes, and smoking (total *R* ^*2*^ **= 0.450). There was a significant association between reactivity to low‐flow and the subsequent FMD response in the overall dataset, and reactivity to low‐flow independently contributed to FMD. These findings suggest that reactivity to low‐flow plays a key role in the subsequent brachial artery FMD response and is important in the interpretation of FMD data.

## Introduction

Hemodynamic wall shear stress influences vascular homeostasis, and participates in the initiation and progression of atherosclerotic cardiovascular disease (CVD) (Davies 2009). It also plays an essential role in modulating vascular endothelial function, influencing the release of endothelium‐derived vasodilators and vasoconstrictors that determine vessel caliber. An alteration to blood flow conditions, like those observed in high‐flow (i.e., reactive hyperemia) and low‐flow states (i.e., cuff‐occlusion), alters wall shear stress with a corresponding change in vessel diameter. During the conventional assessment of endothelial function by brachial artery flow‐mediated dilatation (FMD), different flow conditions occur consecutively, that is, low‐flow to high‐flow (cuff‐occlusion and cuff‐release, respectively). However, it remains uncertain whether there is interplay between vessel reactivity in each of these flow conditions, such that the FMD response is influenced by the preceding vascular response to low‐flow and low wall shear stress. This is an important consideration because a pronounced vasoconstriction during cuff‐occlusion may reduce the subsequent dilatation during FMD, influencing the interpretation of the response.

Previous studies (Gori et al. 2008, 2010) have reported a vasoconstriction in the radial artery during a cuff‐induced low‐flow condition. However, a similar low‐flow condition in the brachial artery results in vasoconstriction in some, vasodilatation in others, and no response in some individuals (Thijssen et al. 2008; Weissgerber et al. 2010; Harrison et al. 2011; Spiro et al. 2011; Rakobowchuk et al. 2012). This nonuniform reactivity to low‐flow has recently been shown to influence the subsequent brachial artery FMD response (Harrison et al. 2011; Spiro et al. 2011; Irace et al. 2016), challenging the interpretation of a diminished FMD response due to aging or the presence of cardiovascular risk factors and/or CVD. For example, a diminished FMD response is typically interpreted as impaired endothelial function (Corretti et al. 2002) or as a result of reduced hyperemic stimulus (Mitchell et al. 2004). However, in healthy cohorts, a diminished FMD response has also been shown to occur if there is reactivity (vasoconstriction) to the low‐flow condition that occurs during cuff‐occlusion prior to FMD. There are few data available to demonstrate whether reactivity to low‐flow is important in data interpretation in clinical populations (Spiro et al. 2011), such as patients with cerebrovascular disease and CVD. In particular, it remains uncertain whether vasoconstriction during low‐flow explains the blunted FMD typically observed in patients with CVD and whether this has clinical relevance.

The aim of this study was to determine if vasoreactivity to low‐flow occurs in a clinical cohort with elevated cardiovascular risk and, if so, whether this influences the magnitude of FMD response in the brachial artery. We also aimed to determine the influence of cardiovascular risk factors and/or CVD, baseline arterial diameter, and estimated hyperemic wall shear stress on the reactivity to low‐flow. We hypothesized that (1) reactivity to low‐flow would be positively associated with the magnitude of FMD response, irrespective of the presence of cardiovascular risk factors and/or CVD, and (2) the magnitude of reactivity to low‐flow would independently contribute to the magnitude of FMD response.

## Material and Methods

### Participants

One hundred and sixty‐five individuals (115 males, 65.8 ± 8.5 years) participated and were allocated to two groups: Increased cardiovascular risk (CVR: *n* = 115, 85 males, 67.0 ± 8.8 years) or healthy control (CTRL: *n* = 50, 30 males, 63.2 ± 7.2 years). Individuals were allocated to CVR if they had previously been diagnosed as having had a stroke/transient ischemic attack, coronary artery disease, peripheral artery disease, type 2 diabetes, hypertension and/or dyslipidemia. Participants in CVR were in a stable condition at the time of the study visit with appropriate medical treatments. Participants allocated to CTRL had no overt CVD, type 2 diabetes, hypertension, or dyslipidemia. In the morning of the study visit, participants were asked to abstain from taking any medications and caffeinated beverages. UK National Research Ethics Service South West Committee approved all study procedures and written informed consent was obtained from all participants.

### Experimental procedures

All experimental procedures were conducted in a temperature‐controlled room. Participants arrived at our department after an overnight fast, had blood samples drawn, consumed a standardized meal and rested supine for 20 min before initiation of the protocol.

Brachial artery reactivity was assessed noninvasively using a reactive hyperemia technique, following established guidelines (Corretti et al. 2002; Thijssen et al. 2011) and previously described by us elsewhere (Gates et al. 2007; Gilchrist et al. 2013; Bond et al. 2015a,b). Briefly, subjects lay supine on an examining bed with the right arm supported and fixed in position using a positioning pillow on a metal table. A small blood pressure cuff was placed around the proximal part of the forearm at least 2 cm below the antecubital fossa. A Doppler ultrasound machine with a multifrequency linear array transducer (SSD‐5500 SV, Aloka, Tokyo, Japan) was used to obtain a two‐dimensional image of the brachial artery. Once the optimal ultrasound image (clear lumen/arterial wall interface) was obtained, the transducer was then clamped using a custom‐made transducer holder to prevent movement.

Baseline brachial artery image and blood velocity were recorded for 60 cardiac cycles. Following the baseline recording, reactive hyperemia was induced by rapidly inflating the forearm blood pressure cuff (AI6, Hokanson, Bellevue, WA) to 250 mmHg to occlude forearm blood flow for 5 min. At 5 min, the cuff was rapidly deflated. Recording of the brachial artery image and blood velocity was restarted 30 sec before deflation and continued until 3 min following deflation.

All brachial artery images and blood velocity (Doppler spectral envelops) were recorded and analyzed by the same investigator using dedicated software (Vascular Research Tools, version 5.8.6, Medical Imaging Applications LLC, Coralville, IA). FMD was calculated as the maximum percentage changes in diameter after cuff‐deflation compared to baseline diameter. Reactivity to low‐flow was calculated as the percentage change in diameter during the last 30‐sec of cuff‐occlusion (during low‐flow) compared to baseline diameter. Reactivity to the low‐flow condition was categorized as vasoconstriction (<0% of baseline diameter), no response (equal to baseline diameter), and vasodilatation (>0% of baseline diameter). Composite vasoreactivity was calculated as the change in diameter from low‐flow to peak FMD diameter, as a percentage of the diameter measured at low‐flow. Shear rate (SR) was estimated by the following equation: (4 × MBV)/diameter (s^−1^), where MBV is mean blood velocity at a respective time point (cm s^−1^). Peak SR and SR difference (baseline SR minus SR during low‐flow) were estimated from the above equation. SR area under the curve until time to peak dilatation (SR aucttp) was also calculated.

### Statistical analysis

All data were presented as means ± SD for variables with normal distribution, and median (interquartile range) for variables with skewed distribution. Categorical variables between CTRL and CVR were compared using a chi‐square test. Independent samples *t*‐tests were used to examine the differences in variables between CTRL and CVR. Pearson's correlation coefficients were used to examine univariate associations between reactivity to low‐flow and the magnitude of FMD response. A stepwise multivariate regression analysis was performed in a pooled dataset to determine the contribution of reactivity to low‐flow and estimated SR indices to the magnitude of FMD response. A log‐transformation was performed for variables with a skewed distribution (e.g., FMD) before statistical analysis. Statistical analysis was performed using IBM SPSS Statistics 22 (IBM, Armonk, NY). Significance was set at *P *<* *0.05. A post hoc power calculation showed that we had 92.7% power to detect a statistically significant association between reactivity to low‐flow and the magnitude of FMD response in this study.

## Results

### Subject characteristics and brachial artery hemodynamic indices

Selected subject characteristics are presented in Table 1. Compared to CTRL, CVR was older and greater in BMI (both *P *<* *0.05). Total and LDL cholesterol were lower in CVR than CTRL (both *P *<* *0.05). HDL cholesterol was lower in CVR than CTRL (*P *<* *0.05). Triglycerides levels and blood glucose concentration were higher in CVR than CTRL (*P *<* *0.05). Blood pressure was similar between the groups. Brachial artery hemodynamic indices are presented in Table 2. All indices were similar between the groups.

**Table 1:** Selected characteristics and medical history of the study participants

	Overall	Control	CVR
Age, years	65.0 (62.0–72.0)	64.0 (60.0–67.0)	66.0 (62.8–74.0)^a^
Sex (M/F), *n*	115/50	30/20	85/30
BMI, kg m^−2^	27.1 ± 4.0	25.0 ± 3.1	28.1 ± 4.0^a^
Total CHOL, mmol L^−1^	4.55 (3.78–5.50)	5.50 (4.95–6.20)	4.10 (3.48–4.80)^a^
LDL CHOL, mmol L^−1^	2.47 (1.77–3.31)	3.29 (2.75–3.91)	2.13 (1.60–2.78)^a^
HDL CHOL, mmol L^−1^	1.43 (1.16–1.75)	1.72 (1.39–2.04)	1.33 (1.09–1.59)^a^
TG, mmol L^−1^	1.03 (0.82–1.53)	0.92 (0.74–1.29)	1.10 (0.85–1.60)^a^
FG, mmol L^−1^	5.25 (4.80–5.70)	5.00 (4.80–5.50)	5.35 (4.70–5.80)^a^
Systolic BP, mmHg	140.7 ± 16.2	139.1 ± 16.3	141.4 ± 16.1
Diastolic BP, mmHg	79.4 ± 8.9	79.1 ± 9.0	79.6 ± 8.92
Stroke/TIA, *n*	89	0	89^a^
CAD, *n*	14	0	14^a^
PAD, *n*	2	0	2
Type 2 Diabetes, *n*	18	0	18^a^
Hypertension, *n*	83	0	83^a^
Dyslipidemia, *n*	92	0	92^a^
Current smoking, *n*	8	2	6
Current Medications			
Biguanides, *n*	14	0	14^a^
Sulfonylureas, *n*	2	0	2
DPP‐4 inhibitors, *n*	1	0	1
Insulin, *n*	1	0	1
* α*‐blockers, *n*	5	1^b^	4
* β*‐blockers, *n*	19	0	19^a^
ACE inhibitors, *n*	41	0	41^a^
ARBs, *n*	13	0	13^a^
CCBs, *n*	29	0	29^a^
Nitrates, *n*	2	0	2
Diuretics, *n*	26	0	26^a^
Statins, *n*	93	0	93^a^
Anticoagulants, *n*	2	0	2
Antiplatelets, *n*	90	1^c^	90^a^
HRT, *n*	1	1	1

CVR, increased cardiovascular risk group; BMI, body mass index; CHOL, cholesterol; LDL, low‐density lipoprotein; HDL, high‐density lipoprotein; TG, triglycerides; FG, fasting glucose; BP, blood pressure; TIA, transient ischemic attack; CAD, coronary artery disease; PAD, peripheral artery disease; DPP‐4, dipeptidyl peptidase‐4; ACE, angiotensin‐converting enzyme; ARB, angiotensin II receptor blocker; CCB, calcium channel blocker; HRT, hormone replacement therapy.

Data are presented as means ± SD or median (interquartile range).

a Significantly different from the control group (*P *<* *0.05).

b Prescribed for benign prostate enlargement.

c prescribed for a condition associated with postorthopedic surgery.

**Table 2:** Selected brachial artery structural and hemodynamic indices of the study participants

	Overall	Control	CVR
D base, mm	3.85 (3.38–4.27)	3.85 (3.06–4.35)	3.90 (3.44–4.26)
D low‐flow, mm	3.80 ± 0.66	3.71 ± 0.76	3.83 ± 0.61
SR diff, sec^−1^	48.6 (34.1–77.3)	45.2 (31.4–72.5)	49.4 (34.6–78.2)
Low‐flow reactivity, %	−0.38 ± 1.59	−0.35 ± 1.69	−0.39 ± 1.55
D peak, mm	3.94 ± 0.64	3.86 ± 0.74	3.98 ± 0.59
FMD, %	3.26 (2.04–4.91)	3.37 (1.83–5.82)	3.12 (2.18–4.80)
Composite reactivity, %	4.07 (2.14–5.21)	4.20 (2.42–5.27)	3.86 (1.93–5.10)
SR peak, sec ^−1^	819.2 (676.5–1021.1)	874.2 (686.9–1053.1)	803.6 (663.0–963.4)
SR aucttp, au	25 559 (21 122–31 760)	24 468 (19 696–29 776)	25 795 (21 675–32 777)
TTP, sec	50.0 (40.0–65.0)	40.0 (35.0–56.3)	50.0 (40.0–70.0)

CVR, increased cardiovascular risk group; D base, baseline diameter; D low‐flow, diameter during low‐flow; SR diff, difference in shear rate; D peak, peak diameter; FMD, flow‐mediated dilatation; SR peak, peak shear rate; SR aucttp, shear rate area under the curve until peak dilatation; TTP, time to peak dilatation.

Data are presented as means ± SD or median (interquartile range).

### Association between reactivity to low‐flow and FMD

**Figure 1:**
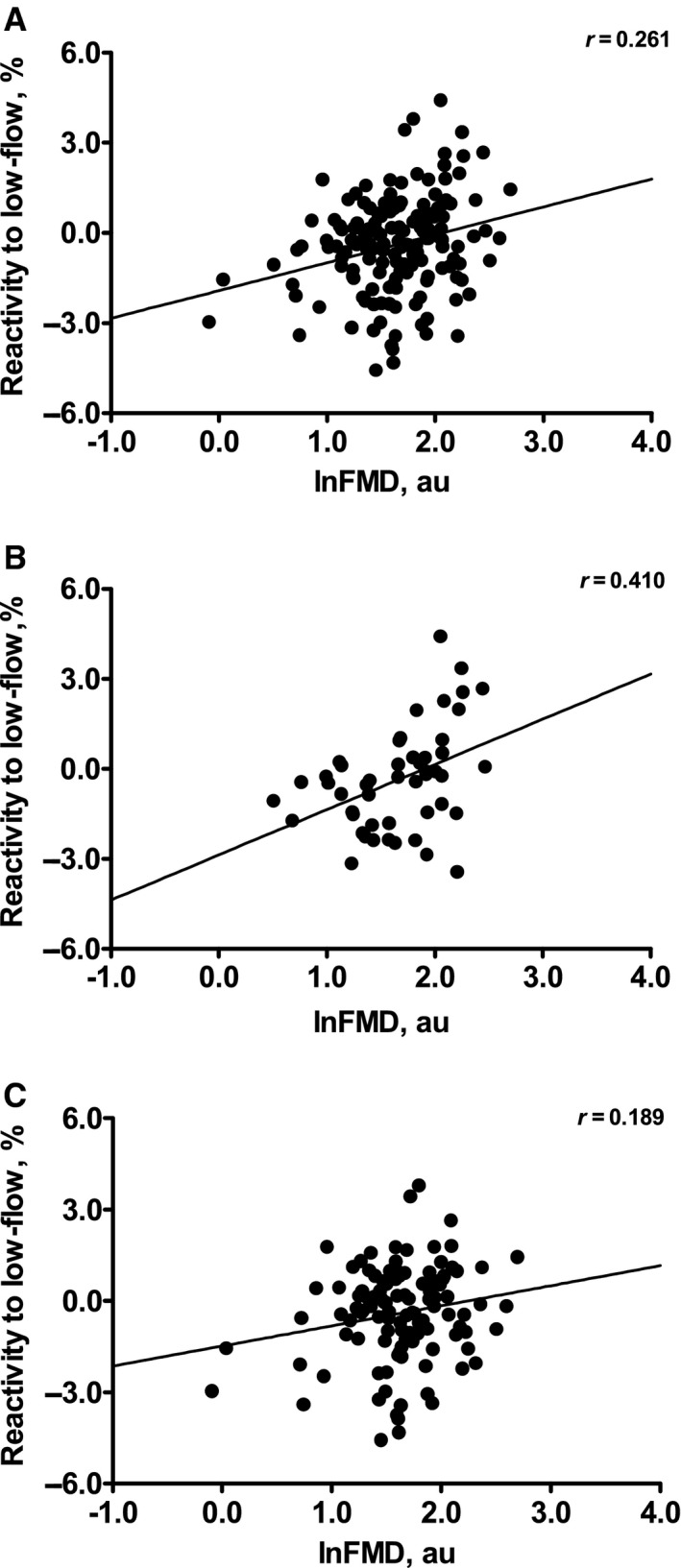
Univariate association between reactivity to low‐flow and FMD in the brachial artery in the overall dateset (A), CTRL (B), and CVR (C). Ln‐ denotes a log‐transformation. Note that a constant (2) was added to each FMD value before log‐transformation to make all the values positive and nonzero.

During the cuff‐induced low‐flow condition, vasoconstriction was exhibited by 95 (58%) out 165 individuals [31 (62%) in CTRL and 64 (56%) in CVR]. In contrast, vasodilatation was exhibited by 68 (41%) of 165 individuals [18 (36%) in CTRL and 50 (43%) in CVR]. Two (1%) of 165 individuals [one (2%) in CTRL and one (1%) in CVR] did not show any response during this time period. Figure 1 shows a univariate association between reactivity to low‐flow and FMD in overall (1), CTRL (2), and CVR (3). There was a significant correlation between FMD and reactivity to low‐flow in all datasets: Overall (*r *=* *0.261), CTRL (*r *=* *0.410) and CVR (*r *=* *0.189, all *P *<* *0.05). Adjustments for age and sex did not alter the observed relationships.

### Contribution of low‐flow reactivity to FMD in the overall dataset

A stepwise multivariate regression analysis was performed in the pooled dataset. When age, sex, current smoking, medical history (the presence of cardio‐ and cerebrovascular disease, hypertension, type 2 diabetes and/or dyslipidemia), systolic blood pressure, estimated SR indices, baseline brachial diameter, and reactivity to low‐flow were included in the model, peak SR (*β *= 0.301), reactivity to low‐flow (*β *= 0.271), and baseline brachial diameter (*β *= −0.454) independently contributed to the magnitude of FMD response (*R* ^*2*^ **= 0.380 for those 3 variables) in addition to sex, the presence of type 2 diabetes, and current smoking (*R* ^*2*^ **= 0.450 for overall).

## Discussion

This study demonstrates that reactivity to low‐flow during cuff‐occlusion influences the magnitude of the subsequent FMD response in the brachial artery in a clinical cohort of middle‐aged and older individuals with increased cardiovascular risk. This association was also observed in healthy middle‐aged and older adults. Reactivity to low‐flow independently contributed to the magnitude of FMD response in the pooled dataset, together with peak SR and baseline diameter. These findings suggest that reactivity to low‐flow plays a key role in the magnitude of FMD response in the brachial artery in patients and in healthy controls.

### Association between reactivity to low‐flow and the magnitude of FMD response

Our data demonstrate that brachial artery reactivity to low‐flow varies considerably between individuals from vasoconstriction to vasodilatation, consistent with previous reports (Weissgerber et al. 2010; Harrison et al. 2011; Spiro et al. 2011; Rakobowchuk et al. 2012). This was the case in the clinical cohort and the healthy control group, and reactivity to low‐flow did not appear to be dependent on cardiovascular risk status: The association between reactivity to low‐flow and the magnitude of FMD response observed in all datasets was unaltered by the presence of cardiovascular risk factors and/or CVD in CVR. These observations suggest that reactivity to low‐flow influences the subsequent FMD response in the brachial artery; that is, a greater vasoconstrictor response during low‐flow results in a smaller vasodilator response after cuff‐deflation and vice versa. Given that this was the case in all groups, reactivity to low‐flow may be considered as one of the key contributing factors for the magnitude of FMD response and an important consideration when interpreting brachial artery FMD data.

The mechanism by which reactivity to low‐flow influences the subsequent magnitude of FMD response remains unknown. It can be speculated that the balance between constitutive vasodilator (e.g., nitric oxide) and vasoconstrictor (e.g., endothelin‐1) substances is altered during low‐flow and the magnitude of this shift influences the extent of vasoconstriction during low‐flow and the subsequent capacity for vasodilatation during hyperemia. We had thought that this might reveal something about the health status of subjects who exhibited vasoconstriction with blunted FMD, but our data showed that this response was no more prevalent in patients than in healthy controls.

Alternatively, vasoreactivity during cuff‐occlusion may be a reflection of the baseline activation state of the endothelium that is revealed during low‐flow, consistent with the suggestion by Gori et al. (2010). Our data indicate that the “resting” level of endothelial activity affects both reactivity to low‐flow and the subsequent FMD response. Activated endothelium (e.g., greater baseline diameter) may show a reduced ability to further vasodilatation in response to a hyperemic stimulus, but may be responsive to vasoconstrictor agents released during low‐flow. In this case, low‐flow may produce vasoconstriction, whereas the subsequent FMD may only be sufficient to return the arterial diameter back toward or just beyond the baseline (activated) diameter. It has also been suggested that the vasoconstrictor agents released during low‐flow are still active at the time of peak dilatation (Weissgerber et al. 2010; Harrison et al. 2011), such that a greater vasoconstrictor response to low‐flow inhibits the magnitude of the subsequent FMD response. Conversely, endothelium in a nonactivated state (e.g., smaller baseline diameter) may show a blunted ability to further vasoconstrict in response to low‐flow, but may respond to a hyperemic stimulus with a marked vasodilatation. Cross‐talk between baseline diameter, reactivity to low‐flow and FMD may therefore be dependent upon the activation status of the endothelium at baseline.

Consistent with this notion, prior isometric handgrip exercise caused an increase in baseline radial artery diameter, a greater reactivity to low‐flow, and a diminished FMD response compared to the no‐prior exercise condition (Gori et al. 2010). A greater baseline brachial diameter, vasoconstriction during low‐flow and diminished FMD were also demonstrated following percutaneous coronary intervention compared to the preintervention condition (Spiro et al. 2011). In both of these studies, the composite vasoreactivity (the change in diameter from the low‐flow condition to FMD) was similar between the resting and postintervention conditions, indicating the need to include the low‐flow arterial diameter in the interpretation of the data.

Some participants exhibited vasodilatation in response to low‐flow. A vasodilatory response to low‐flow (and low wall shear stress) is counterintuitive, but is consistent with previous observations (Thijssen et al. 2008; Weissgerber et al. 2010; Harrison et al. 2011; Spiro et al. 2011; Rakobowchuk et al. 2012). Thijssen et al. (2008) have postulated that local arterial pressure changes immediately proximal to the occluding cuff may alter transmural pressure gradients (Laughlin et al. 2008), inducing changes in vasodilatation.

### Contribution of reactivity to low‐flow for the magnitude of FMD response in the overall dataset

We used a stepwise multiple regression analysis to determine the variables that independently contribute to the FMD response in the overall dataset. We found that reactivity to low‐flow, peak SR, and baseline brachial artery diameter independently contributed to the magnitude of FMD response along with sex, the presence of diabetes, and current smoking. This study is the first, to our knowledge, to demonstrate significant contributions from reactivity to low‐flow in addition to baseline brachial artery diameter and hyperemic SR to the magnitude of FMD response in middle‐aged and older individuals with diverse cardiovascular risk profiles. Baseline diameter is a recognized strong predictor for the magnitude of FMD response (Herrington et al. 2001; Silber et al. 2005; Maruhashi et al. 2013) as well as a predictor for CVD events in the elderly (Yeboah et al. 2007). Thus, our finding supports an association previously observed between baseline brachial diameter and the magnitude of FMD response. Importantly, reactivity to low‐flow and peak SR also independently contributed to the magnitude of FMD response in this study. Baseline diameter, peak SR, and reactivity to low‐flow are each influenced by different factors: Baseline diameter being influenced by resting endothelial tone (Gori et al. 2008, 2010), peak SR being influenced by forearm microvascular function (Mitchell et al. 2004; Anderson et al. 2011), and reactivity to low‐flow is postulated to be influenced by endothelium‐mediated vasodilator as well as vasoconstrictor substances (Gori et al. 2008). In other words, the magnitude of FMD response may be a consequence of (1) endothelial activation at the time of assessment, (2) forearm microvascular function, and (3) the balance between vasodilator and vasoconstrictor substances during low‐flow. We speculate that in a population with diminished endothelial function due to aging, the presence of cardiovascular risk factors and/or CVD, these three hemodynamic factors may in part determine the magnitude of FMD response in the brachial artery.

### Implications from this study

Our observation that a greater vasoconstriction during low‐flow produces a smaller FMD response indicates the need to measure reactivity to low‐flow as part of the FMD procedure. There could be a potential role for reactivity to low‐flow to be a marker of endothelial function individually or in combination with the magnitude of FMD response (Irace et al. 2016). Reduced brachial artery FMD itself is a well‐known marker of endothelial dysfunction as well as a strong predictor of future CVD events (Green et al. 2011). However, it is currently unknown (1) whether the vasoconstriction during low‐flow itself is a marker of endothelial function, and (2) whether the composite vasoreactivity is a more precise indicator of endothelial function and accordingly more accurately predicts future CVD events than the conventional FMD does. Follow‐up on the current cohort is planned to explore if the composite vasoreactivity is a useful predictor for future CVD events.

### Limitations

We were unable to assess brachial artery stiffness which is reported to be positively associated with the magnitude of reactivity to low‐flow in a population with variable coronary artery disease risk factors (Harrison et al. 2011). We did not assess endothelium‐independent dilatation using an exogenous nitric oxide donor (e.g., nitroglycerin). However, a recent study has demonstrated an endothelium‐dependent nature of reactivity to low‐flow in the radial artery with transradial catheterization (Dawson et al. 2012), which in part supports our findings. Finally, current medication usage in CVR could have influenced our brachial artery hemodynamic indices. However, these indices were similar between groups after statistical adjustment for medication usage. A previous study has also shown that acute vasoactive drug administration had no effect on brachial artery reactivity in healthy individuals or coronary artery disease patients (Gokce et al. 2002).

## Conclusion

This study demonstrated that reactivity to low‐flow was associated with the magnitude of FMD response in a clinical cardiovascular risk cohort, a healthy middle‐aged and older cohort and in the pooled dataset of both these cohort. Furthermore, reactivity to low‐flow independently contributed to the magnitude of FMD response in the pooled dataset in addition to already known contributors (baseline diameter and peak SR). These findings suggest that in the brachial artery, reactivity to low‐flow plays a key role in the subsequent FMD response in both the cohort with diverse cardiovascular risk and in the healthy control group.

## Conflicts of Interest

Nothing to declare.
